# Nitrous Oxide Use Precipitates Pulmonary Embolism: A Case Report

**DOI:** 10.7759/cureus.69347

**Published:** 2024-09-13

**Authors:** Nancy Nguyen, Jessica Cao, Deborah Carlson, Lynn Kong, Graal Diaz

**Affiliations:** 1 Medicine, Community Memorial Health Systems, Ventura, USA; 2 Medicine, Western University of Health Sciences, Pomona, USA; 3 General Surgery, Community Memorial Hospital, Ventura, USA; 4 Graduate Medical Education / Internal Medicine, Community Memorial Hospital, Ventura, USA; 5 Department of Oncology, Ventura Cancer Center, Ventura, USA; 6 Graduate Medical Education, Community Memorial Hospital, Ventura, USA

**Keywords:** case report, b12 supplementation, b12 deficiency, nitrous oxide, hyperhomocysteinemia, venous thromboembolism, whippets

## Abstract

Nitrous oxide (N2O) has a lengthy history of use as an anesthetic and has recently found popularity as a recreational euphoric hallucinogen. The odorless, colorless, non-flammable gas interferes with Vitamin B12 resulting in a cascade of effects, including hyperhomocysteinemia. It has long been proposed that hyperhomocysteinemia adversely affects the cardiovascular system, producing atherogenic and prothrombotic diseases.

In this case vignette, we describe a case in which a healthy patient presented with venous thromboembolism (VTE) that we suspect could have been precipitated by daily and significant recreational use of N2O. Anticoagulation therapy was given, and there was a significant improvement in the pulmonary emboli. As recreational use of N2O increases, it is essential to recognize that hyperhomocysteinemia may also produce a thrombotic state.

## Introduction

Nitrous oxide (N2O) has been used as an anesthetic since 1844 in surgery and dentistry. There is usually a subsequent rise in homocysteine levels. The proposed mechanism is inhibition of folate metabolism and deoxyribonucleic acid synthesis [1,2]. Inhibition of homocysteine to methionine conversion in the DNA synthesis pathway results in an elevation in homocysteine levels. Exposure to N2O after four hours of anesthesia showed a statistically significant increase in the level of plasma homocysteine. The homocysteine concentration is greater given a longer duration of anesthesia [3]. Increased homocysteine levels are associated with endothelial dysfunction [4] and atherosclerotic disease [5].

Hyperhomocysteinemia can predispose patients to a hypercoagulable state and increase the risk of arterial atherogenic disease and venous thrombosis [6]. The relationship between hyperhomocysteinemia and the cause of venous thromboembolism (VTE) remains a subject of debate [7]. A meta-analysis assessed hyperhomocysteinemia as a risk factor for VTE, highlighting that individuals under 60 years of age are at a markedly increased risk for VTE. Overall, hyperhomocysteinemia has been associated with VTE in some but not all studies [8]. We present a unique case of a 42-year-old patient with VTE secondary to the recreational use of nitrous oxide.

## Case presentation

A 42-year-old patient presented to the emergency department with a two-day onset of dyspnea, pleuritic chest pain, shortness of breath, and back pain. It was worse with movement and limited daily activities. The patient denied recent trauma or falls. She reported a recent increase in N2O use, with her last use occurring two days prior. The past medical history was notable for anxiety and substance abuse with N2O. The patient had recreational N2O use of five to ten boxes per day, each containing 24 canisters (120 - 240 whippets per day) for the past year.

**Figure 1:**
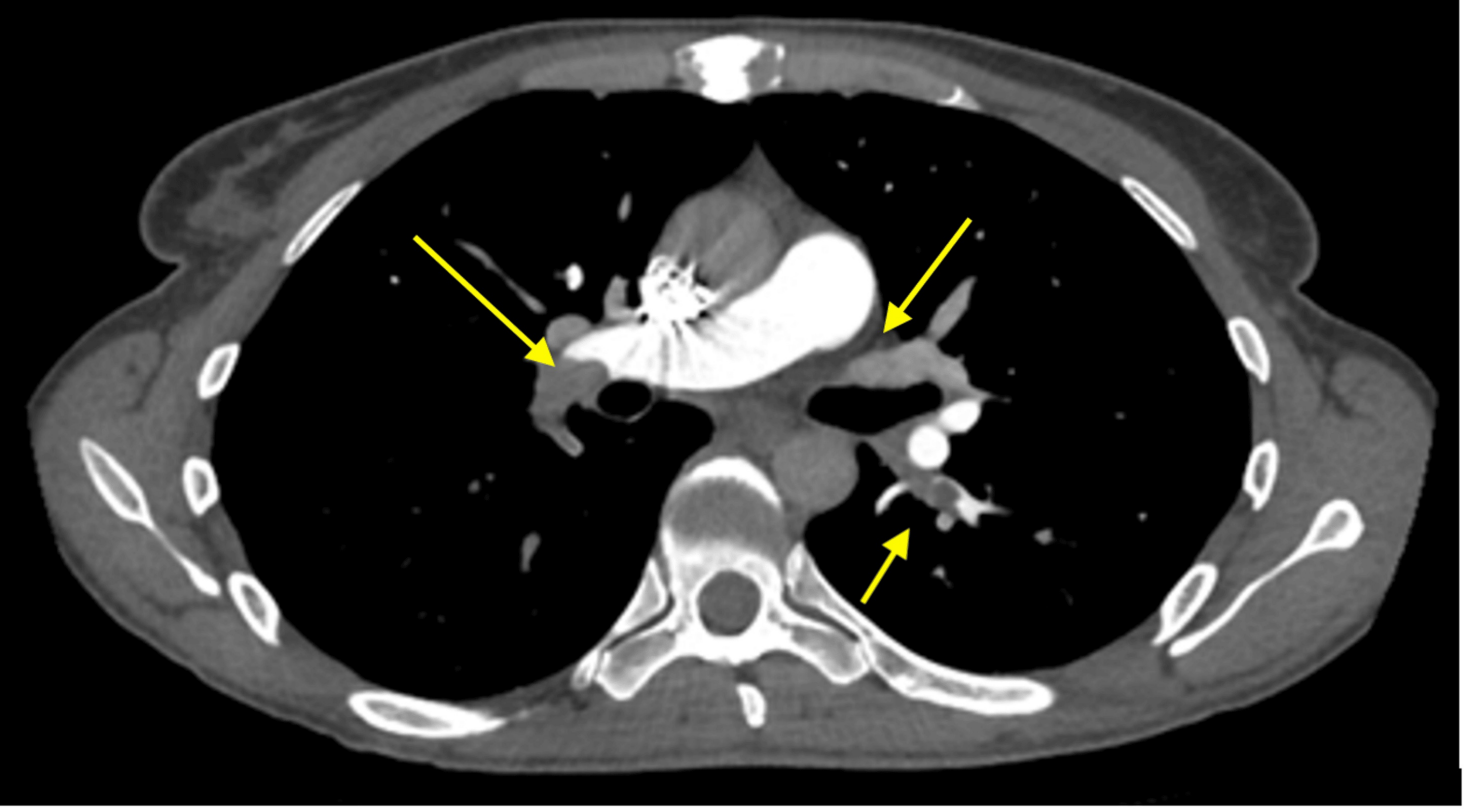
Helical computed tomography pulmonary angiography showing bilateral pulmonary embolism

On admission the vital signs were within normal limits: pulse was 76 beats per minute, and oxygen saturation was 100% on room air. The physical examination demonstrated an anxious-appearing patient, normal cardiopulmonary findings, and no neurological deficits. The initial laboratory studies, ECG, and chest radiograph were unremarkable. The patient did not present with leg swelling, arrhythmia, or other signs that might typically prompt further investigation of potential embolic origins. Given the clinical presentation and findings, the pulmonary embolism was most likely a de novo event, arising directly within the pulmonary circulation without an identifiable source in the limb or abdominal vessels. Although the Well's criteria indicated a low pre-test probability, the D-dimer was significantly elevated at 1,808 ng/ml (normal <250 ng/ml). Computed tomography pulmonary angiography (CTPA) revealed acute, extensive pulmonary emboli throughout the bilateral lower lobe pulmonary arteries, with additional involvement in the right middle lobe and left lingular pulmonary arteries (Figure 1). Both homocysteine and cyanocobalamin levels were elevated, while routine blood tests, as well as hepatic, renal function, and folate levels, remained within normal limits (Table 1).

**Table 1:** Patient’s laboratory values upon admission ^1 ^nanograms per milliliter^, 2^ micromoles per liter^, 3 ^milliequivalents per liter^, 4^ milligrams per deciliter^, 5^ thousands per microliter^, 6 ^grams per deciliter

Parameter	On admission	Reference range
D-Dimer	1,808 ng/mL^1^	0-308 ng/mL^1^
Prothrombin time (PT)	13.8 seconds	9.8 - 13.0 seconds
INR	1.32	0.8-1.2
Homocysteine	18.6 uM/L^2^	5-15 uM/L^2^
Cyanocobalamin	1158 ng/mL^1^	160-950 ng/mL^1^
Sodium	134 mEq/L^3^	136-148 mEq/L^3^
Potassium	3.8 mEq/L^3^	3.5 -5.0 mEq/L^3^
Chloride	105 mEq/L^3^	96-112 mEq/L^3^
CO_2_	21 mEq/L^3^	23-30 mEq/L^3^
BUN	11 mg/dL^4^	7-22 mg/dL^4^
Creatinine	0.79 mg/dL^4^	0.5-1.2 mg/dL^4^
Glucose	104 mg/dL^4^	70-100 mg/dL^4^
WBC	8.2 K/uL^5^	4.8-10.8 K/uL^5^
Hemoglobin	14.3 g/dL^6^	4.20-5.40 g/dL^6^
Hematocrit	42.1%	37.0-47.0%
Platelet count	198 K/uL^5^	130-400 K/uL^5^

The patient was admitted to the hospital and started on anticoagulation with therapeutic doses of low-molecular-weight heparin and warfarin. Her length of stay at the hospital was five days. She was treated with therapeutic doses of enoxaparin sodium for five days and warfarin, initially 5 mg and then 3 mg for bridge. The international normalized ratio (INR) was therapeutic on hospital day four and was supratherapeutic on hospital day five. The patient’s warfarin was held on the day of discharge and restarted the next day at 3 mg. Anticoagulation follow-up was arranged outpatient and a lab slip was issued for prothrombin time and international normalized ratio (PT/INR) check. Further workup for the patient's hypercoagulable state was recommended for when she discontinues anticoagulation therapy in three to six months.

**Figure 2:**
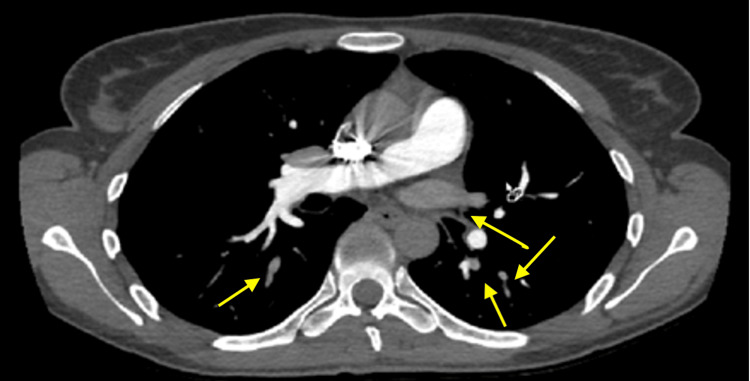
Helical computed tomography pulmonary angiography demonstrating the resolution of embolism in the right pulmonary artery

Our patient was re-presented to the emergency department with dyspnea two months later. Although therapy adherence at home is unknown, a repeat CTPA demonstrated significant improvement of the pulmonary emboli (Figure 2), and our patient was referred for outpatient evaluation of symptoms. 

## Discussion

The recreational abuse of N2O has been shown to be associated with a rise in severe thrombotic events. Recently, N2O has found a new venue as a recreational drug. N2O, often inhaled through small canisters, where it is stored as a whipping agent for making cream or "whippets," is abused not only for its analgesic properties but also for its euphoric effects [9]. 

In this case presentation, we identify the provoking factor for pulmonary embolism to be likely due to the patient's N2O usage. The patient had been inhaling N2O from a whipped cream bottle two days prior to the onset of symptoms. The patient denied any other possible contributing factors to a hypercoagulable state including a history of malignancy, endocrine disorders, infection, and/or autoimmune conditions. Nor did the patient’s clinical presentation or laboratory results indicate an obvious source for thromboembolism, except for hyperhomocysteinemia. Therefore, we hypothesize that the patient’s elevated homocysteine level was associated with her excessive recreational N2O use as has been concluded in previous similar case studies.

The conversion of homocysteine to methionine relies on vitamin B12 and folic acid as cofactors. Deficiency of either vitamin B12 or folic acid can cause elevated homocysteine levels (Figure 1) [10]. The serious adverse effects of N2O primarily result from the irreversible oxidizing of the cobalt atom in the B12 vitamin, inhibiting methionine synthase and its pathways [11], thereby leading to a buildup of homocysteine in the blood. One case report stated that a 21-year-old female with no medical history presented to the emergency department for confusion, hallucinations, weakness, and falls. She was accompanied by her roommates, who endorsed that the patient was abusing N2O at home. The patient was found to have elevated serum homocysteine and methylmalonic acid levels but normal vitamin B12 and folate levels. Genetic testing showed a homozygous methylenetetrahydrofolate reductase (MTHFR) polymorphism. MTHFR is documented as a known cause of hyperhomocysteinemia, which can explain the elevated homocysteine level in this patient [11]. Elevated homocysteine levels can also be caused by various disease states including diabetes mellitus, cancer, reduced thyroid function, lupus, and inflammatory bowel disease. Additionally, specific medications like cholesterol-lowering agents, metformin, methotrexate, anticonvulsants, theophylline, and levodopa can also lead to increased homocysteine levels [12].

In this case, potential sources for hyperhomocysteinemia as described above were ruled out, therefore leading to the conclusion that N2O abuse was the cause of hyperhomocysteinemia and ultimately pulmonary embolism. There are many other documented cases of thromboembolic events in patients with similar histories and all had recreational N2O use with elevated homocysteine levels. In one case, a 16-year-old female was admitted to the emergency department with symptoms of headache, lethargy, nausea, and vomiting, culminating from excessive N2O abuse over several weeks. Brain imaging confirmed extensive cerebral venous thrombosis, a rare and serious condition. This case presented a significantly elevated homocysteine level without other identifiable vascular risk factors leading to a conclusion that N2O was the culprit in creating a hypercoagulable state [13]. 

In another case report, a 20-year-old male had a history of repetitive seizures and N2O inhalation over the past three months. The imaging revealed venous hemorrhagic infarction of the left temporal lobe secondary to thrombosis of the sagittal, left transverse, and sigmoid sinuses. The elevation in serum homocysteine levels, with levels of serum vitamin B12 and folic acid falling within normal ranges, suggested a discrepancy between the elevated homocysteine and the seemingly adequate levels of the vitamins crucial for homocysteine metabolism [14].

There have also been cases where elevated homocysteine levels were not seen in patients abusing N2O yet a thromboembolic event had occurred with other causes being ruled out. For example, one case report of a 25-year-old high school graduate who had been abusing N2O for 20 months was found to have normal blood vitamin B12, folate, homocysteine, and beta-human chorionic gonadotropin (beta-hCG) levels. Head CT showed hemorrhagic infarction and subarachnoid hemorrhage. MR angiography and venography were normal. Head MRI identified left frontal isolated cortical vein thrombosis. Therefore, the presence of hyperhomocysteinemia as an indicator of N2O abuse causing thrombosis isn't consistent in all instances [15].

A case series showed that between January 2015 and May 2021, 326 patients reported recreational use of N2O. Seventeen patients presented with severe thrombotic events associated with N2O. These cases involved both arterial (30%) and venous thrombosis. Five patients presented with arterial thrombosis and 12 patients presented with VTEs (10 with pulmonary embolisms, one with portal vein thrombosis, and one with cerebral vein thrombosis). Additionally, homocysteine concentrations were severely increased [16].

In cases where hypercoagulability due to hyperhomocysteinemia occurred, it was rarely presented as an acute pulmonary embolism. Like our case report, a 29-year-old male with long-term N2O abuse sought evaluation for acute chest pain. His diagnostic workup revealed pulmonary embolism, deep vein thrombosis, and hyperhomocysteinemia. Again, there was a potential correlation between N2O abuse and VTE due to its impact on homocysteine levels. Hyperhomocysteinemia has been suspected to cause endothelial dysfunction, platelet and clotting activation, and impaired fibrinolysis. The report suggests a possible mechanism where chronic N2O use leads to decreased active vitamin B12 levels, causing hyperhomocysteinemia and subsequent thrombosis. The cessation of N2O usage in this case report correlated with normalization of homocysteine levels and improvements in thrombotic conditions [17].

The patient reported that her past medical history was notable for anxiety. The patient also reported no prescription or over-the-counter medication use besides B12 supplements and N2O. According to the follow-up progress notes, the patient did not have recurrent VTE after stopping N2O. Pregnancy, infection, malignancy, and prescription medications were ruled out as the source of a hypercoagulable state.

We can logically conclude from this case study that there is a link between N2O abuse and thrombotic events. We cannot determine the exact amount of N2O use that causes this relationship. It can also be stated that elevated homocysteine levels are a strong indicator of N2O abuse and thromboembolic risk. Though this case report and others mentioned above support these statements, further outpatient hematologic testing would be needed in this case to definitely rule out another cause of thrombosis such as protein C and/or S deficiency, antithrombin deficiency, prothrombin mutation, factor V Leiden mutation, or other coagulation disorders. The lack of these tests is a significant limiting factor in this case report. 

## Conclusions

Recreational drug use, particularly the inhalation of N2O, poses a growing public health concern due to its potential to disrupt critical metabolic pathways. This case highlights the significant risk of VTE associated with chronic N2O abuse, likely mediated through the pathway of hyperhomocysteinemia. Given the increasing prevalence of N2O use, healthcare providers should maintain a high index of suspicion for VTE in patients presenting with a history of N2O abuse. Chronic N2O abuse should be recognized as a potential risk factor for VTE due to its interference with folate metabolism and subsequent hyperhomocysteinemia. Early identification and intervention are critical to preventing severe thrombotic events in this population.

Future research should focus on establishing a clear causal relationship between chronic N2O use and the development of VTE through large-scale, longitudinal studies. Additionally, investigating the dose-response relationship between N2O exposure and the severity of hyperhomocysteinemia and thrombotic outcomes could provide valuable insights. Preventive strategies, such as folate supplementation for at-risk individuals, should also be explored to mitigate the risk of thrombotic complications. Finally, the effectiveness of public health interventions aimed at reducing recreational N2O use and its associated health risks warrants further assessment.
